# Proceedings of the Union Medical Association

**Published:** 1857-11

**Authors:** Thomas Wilkins, S. S. Condon

**Affiliations:** Pres’t.; Sec’y pro tem.


*Proceedings of the Union Medical Association.*

Pursuant to adjournment, and in accordance with previous notice, publicly given by the Corresponding Secretary, the Union Medical Association convened at the Masonic Hall, in the city of Jonesboro, on Tuesday morning, Nov. 10, 1857.

The Association was called to order by Thomas Wilkins, M.D., President.

On motion of Hugh McVean, M.D., of Anna, Ill., Dr. S. S. Condon was elected Secretary *pro tem.*

Thereupon, on motion of Dr. A. D. Stearns, of Vandalia, Hugh McVean, M.D., was unanimously elected a member of this Association.

On motion of Dr. McVean, Drs. H. C. Hacker, MM. Goodman and S. C. Toler, of Jonesboro, were each unanimously elected as members.

On motion of S. S. Condon, Dr. Roberts, of Carbondale, was unanimously elected a member.

By order of the President, the roll was then called, and the following gentlemen answered, viz: Drs. Dunning, of Centralia; Haller, Stearns and Wilkins, of Vandalia. The absentees were noted by the Secretary.

On motion of H. McVean, James V. Brooks, M.D., of Jonesboro, was also duly elected a member of this Association.

On motion of S. C. Toler, George Bratton, M.D., of Vienna, was likewise duly elected a member.

On motion of Dr. Dunning, J. W. White, M.D., of Central City, was duly elected a member.

S. S. Condon, M.D., Chairman of the Committee of Arrangements, made report, that the Masonic Hall was obtained for, and was at the service of the Union Medical Association, which report was, on motion of Dr. Stearns, accepted.

On motion of Dr. Stearns,

*Resolved*, That Dr. William White, of Salem, is charged with holding and refusing to pay over certain moneys belonging to this Society. The Secretary is instructed to notify him of said charge, and request him to appear and answer at the next meeting.

On motion of Dr. Haller, Dr. Lucas, Treasurer of this Society, be instructed to call upon Dr. William White, of Salem, and receive from him all moneys in his hands belonging to this Society, and that he receipt Dr. White therefor, in the name of the Society.

On call of the President, Dr. Haller, of Vandalia, read in a clear and impressive manner, a very able paper on "Retention of the Placenta from Morbid Adhesions," which elicited some discussion, which was participated in by Drs. Roberts, McVean, Condon and Haller; and on motion of Dr. Dunning, said paper was received and ordered to be filed upon the records.

On call of the President, Dr. Stearns then read before the Society an exceedingly valuable, interesting and eloquent paper on the "Physiological, Pathological and Therapeutical Effects of Sulphate of Quinine."

On motion of Dr. Condon, the Society then adjourned until 8 o'clock Wednesday morning.

WEDNESDAY MORNING, NOVEMBER 11, 1857.

The Society met, pursuant to adjournment; present as yesterday.

By order of the President, the minutes of the last meeting, held at Richview, were read by the Secretary, and approved by unanimous vote of the Society.

Dr. Bundy, Corresponding Secretary, presented for the consideration of the Association, a short communication to him from Dr. White, of Salem, which, upon motion, after debate, was laid upon the table.

Dr. Condon introduced the following resolution, which will come up at the next regular meeting:

*Resolved*, That all regular members of the legal profession, of moral character, be permitted and invited to become full members of this Association.

Adjourned to 2 o'clock P. M.

2 O'CLOCK P. M.

Society met pursuant to adjournment.

On motion of Dr. Bundy,

*Resolved*, That the Union Medical Association hold its next regular meeting at Centralia, Illinois.

Dr. Bundy gave notice that at the next meeting he should move to so amend the Constitution as to allow the Society to meet as often as wished for in one place.

On motion of Dr. McVean,

*Resolved*, That we memorialize the next Legislature to pass a law prohibiting persons from collecting fees for medical services, who were not graduates of some respectable medical school.

On motion of Dr. Dunning,

*Resolved*, That a committee of three be appointed to petition the Legislature, or by any other means, to procure the passage of a law to prevent incompetent persons from collecting fees for any medical services.

Drs. Dunning, Goodman and Toler were appointed said committee.

Dr. Dunning proposed J. B. Lamb, M.D., of Centralia, as a member of this Society, who thereupon was duly elected.

Drs. McCord and Lamb, of Centralia, and J. W. White, of

Central City, were appointed a Committee of Arrangements for the next meeting.

Dr. McVean was appointed to write an *Essay Medical* for next meeting; and Dr. S. S. Condon to prepare and deliver an address.

On motion of Dr. Bundy, Drs. Hotchkiss and Murphy were fined five dollars each, for failing to deliver addresses at this meeting.

On motion of Dr. Wilkins, (Hacker in the chair,) the thanks of this Society are gratefully tendered the Committee of Arrangements, Drs. Condon, Brooks and Hacker, for the satisfactory manner in which they have discharged their duties, and to professional brethren of Jonesboro, for the cordial kindness with which we have been treated while in attendance at this Association, and to the citizens of Jonesboro for the hospitalities extended to us—the remembrance of which will be ever gratefully cherished.

On motion of Dr. Condon, adjourned until the next regular meeting.

THOMAS WILKINS, M. D., *Pres't.*

S. S. CONDON, M. D., *Sec'y pro tem.*

